# AI-enabled photoresponsive nanoplatforms for coordinated osteosarcoma therapy and bone regeneration

**DOI:** 10.3389/fbioe.2026.1813288

**Published:** 2026-03-23

**Authors:** Qinghan Li, Shuo Duan, Minglei Zhang

**Affiliations:** Department of Xinmin Orthopedic, China-Japan Union Hospital of Jilin University, Changchun, China

**Keywords:** artificial intelligence, immunomodulation, nanoplatforms, osteosarcoma, phototherapy

## Introduction

Osteosarcoma is one of the most common primary malignant bone tumors, which mainly occurs in children and adolescents. Although surgical resection, combined with chemotherapy and radiotherapy, has improved the local control rate to some extent, the improvement of long-term survival rate is still limited for patients at risk of metastasis or recurrence. In addition, treatment is often accompanied by serious complications, such as bone structure destruction and functional loss (Su et al., 2025). Although the current standard treatment scheme has made progress in inhibiting the primary focus, the difficulty in repairing the large bone defect after operation, the tumor-related immunosuppression microenvironment and the persistent risk of recurrence remain urgent and complex clinical challenges (Dong et al., 2025).

With the development of nanotechnology, nano-enabled methods have become a multidisciplinary strategy, which has great potential in tumor treatment. Through precise targeted delivery and stimulus-responsive release mechanisms, nanocarriers can increase the accumulation of drugs in tumors, improve the local therapeutic effect, reduce systemic toxicity and reduce the risk of multidrug resistance (Kortam et al., 2024). On this basis, scientists have developed photoresponsive nanoplatforms, which can realize controlled local drug release or photothermal effects under external light irradiation. Importantly, these platforms can also activate signal pathways related to bone matrix repair at the same time, thus inhibiting tumor growth and promoting bone regeneration (Byun et al., 2026). This “dual-functional” strategy, which combines anti-tumor activity and osteogenic support, shows advantages in preclinical research, and provides a new idea for simultaneously eliminating residual tumor cells and reconstructing bone function.

However, although nanoscale delivery system we designed is becoming increasingly complex and sophisticated in structure and function, a key problem has not been completely solved: they are not “intelligent and coordinated”. Specifically, most of the current systems work according to preset parameters, and there is no way to adjust the intensity of treatment and promote the pace of tissue regeneration according to the dynamic changes of tumor microenvironment, which limits the personalized optimization for different patients and different stages of illness. In recent years, scientists have studied the application of artificial intelligence in nano-medicine, and found that artificial intelligence (AI) is valuable in optimizing the design of nano-carriers, predicting drug release behavior and helping to evaluate therapeutic efficacy, which provides a technical basis for developing highly controllable intelligent nano-therapy systems. Therefore, if AI is taken as a core module and added to the photoresponsive nano-delivery platform, and it is responsible for dynamic decision-making and parameter adjustment, a new strategic framework may be formed. This framework can adaptively balance tumor treatment and tissue regeneration, and provide theoretical and technical support for accurate treatment of osteosarcoma and functional reconstruction.

**FIGURE 1:**
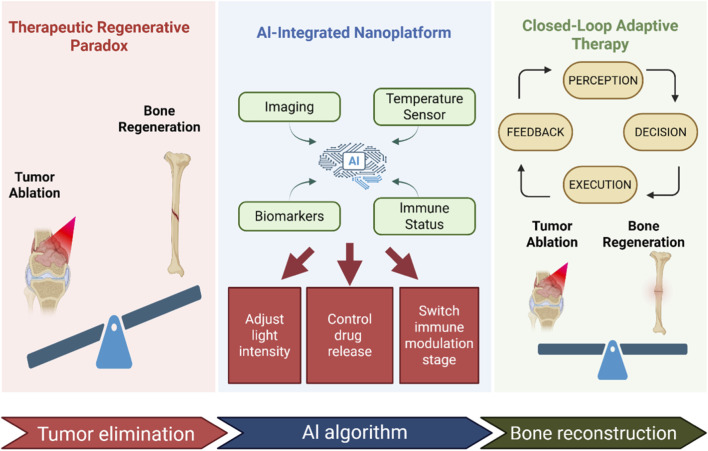
Schematic illustration of an AI-driven photoresponsive nanoplatform for closed-loop coordination of osteosarcoma therapy and bone regeneration.

In this framework, AI has three complementary functions. First of all, it helps to predict and optimize the design of nanocarriers and find out the relationship between structure and function. Secondly, it assists treatment planning through multimodal data modeling. Thirdly, in the implementation stage, it can dynamically adjust irradiation parameters and drug release according to real-time biofeedback, and realize adaptive closed-loop regulation. This layered function distinguishes AI integration from traditional static nano-platform design. The conceptual architecture and operational logic of this integrated strategy are synthesized in Figure 1. By transitioning from a “static-preset” to an “adaptive-feedback” paradigm, this framework aims to resolve the inherent competition between aggressive tumor ablation and delicate bone microenvironment preservation. In the following sections, we will first dissect the fundamental therapeutic paradoxes in osteosarcoma management and then detail how AI-driven decision-making facilitates the transition toward precision functional reconstruction.

## Biological and therapeutic paradox of osteosarcoma treatment and bone regeneration

The main goal of treating osteosarcoma is to completely eliminate tumor cells and reduce recurrence and metastasis. But tumor ablation may destroy the local microenvironment needed for bone repair. Hyperthermia and reactive oxygen species can induce apoptosis or necrosis of tumor cells, but may also inhibit the proliferation and osteogenic differentiation of osteoblasts (Xie et al., 2024). Experiments and transformation studies show that this stress state will destroy the osteogenic regulatory network, change the metabolic balance of cells and reshape the interaction between local immunity and bone microenvironment (Tian et al., 2023). Consequently, aggressive tumor ablation may inadvertently compromise regenerative potential, underscoring the biological coupling between therapeutic intensity and tissue repair capacity. In near-infrared phototherapy, high-intensity irradiation will quickly raise the local temperature to the level of cytotoxicity, but even low-intensity irradiation may damage the regenerative steady state if it takes too long or is not properly controlled. Therefore, photothermal monotherapy has formed a time-and dose-dependent contradiction between tumor clearance and bone regeneration (Yu et al., 2023).

There is also complex immune cell infiltration and signal crosstalk in the microenvironment of osteosarcoma, and these dynamic changes will affect the therapeutic effect and tissue repair in both directions. Although immunomodulation based on nanocarriers can reshape immunosuppression to enhance anti-tumor activity, over-activation of immunity may damage immune support for bone formation and reconstruction (Tian et al., 2023). Therefore, achieving stage-specific immune transformation and precise control is the key to coordinated treatment.

The aim of bifunctional nanomaterials is to combine powerful anti-tumor effects with osteogenic support through biological activity signals or mechanical scaffolds. But these two functions often “fight” in the same space-time environment. For example, while simultaneously inhibiting tumor growth, which makes it difficult for static design to maintain the dynamic balance of “treatment-regeneration” (Byun et al., 2026). Therefore, under the sequential mode of “treating tumor first, then repairing”, a key problem is how to ensure the complete elimination of tumor and minimize the damage to osteoblasts and their regenerative microenvironment. This reflects the fundamental contradiction between anti-tumor intensity and regenerative potential.

## AI-driven dynamic optimization framework for coordinated osteosarcoma treatment and bone regeneration

Real treatment-regeneration coordination requires not only an efficient photoresponsive nano-platform, but also an intelligent decision-making system that can adjust parameters according to biofeedback. The combination of AI and nano-medicine is changing nano-delivery from static design to data-driven, real-time optimization and adaptive execution, thus providing a new strategy for collaborative control and bone regeneration of osteosarcoma (Chou et al., 2025; Guan et al., 2025).

In the design of nano-drugs, AI models can capture the nonlinear relationship between the physical and chemical characteristics of nano-carriers and *in vivo* behavior, and support predictive design and parameter optimization. Supervised learning can find out the characteristics of promoting tumor targeting and efficient delivery from a large number of nanoparticle data, and reduce trial and error. Deep learning and reinforcement learning can simulate the carrier-microenvironment interaction and guide the structure integration (Samathoti et al., 2025). AI can also simulate pharmacokinetics and biological distribution, detect toxicity and off-target risk in advance, and help screen safer multifunctional carriers.

In order to overcome the limitation of only doing one thing, AI can now carry out multi-objective optimization and find the best balance between several constrained performance goals. For example, the algorithm can find ways to maximize photodynamic energy conversion and drug accumulation in tumors, and at the same time control drug release rates to avoid impairing osteoblast function, so as not to affect osteoblasts. They can also find a suitable time window to switch between pro-inflammatory signals and anti-inflammatory signals, so that the steps of immunomodulation and regeneration and repair can be coordinated. The multimodal model, which combines genomics, imaging and histology, can further help to design the carrier and parameter scheme for specific patients and find the best balance between anti-tumor effect and bone regeneration (Guan et al., 2025). For example, we can establish a constrained optimization model, with the goal of making photothermal ablation of tumors the best, while ensuring that the expression of osteogenic markers is not lower than the safety standard. This quantitative trade-off model allows us to adjust the intensity of treatment without affecting the ability of bone regeneration.

At the executive level, AI can establish closed-loop control with photoresponsive nanocarriers. By monitoring the changes of tumor size, local temperature and biomarkers, the algorithm can adjust the light intensity, duration and frequency, control drug release, and continuously evaluate the treatment effect and repair response. This closed-loop system will constantly optimize the operation according to the actual feedback, instead of rigidly following the preset scheme, which may improve drug accumulation at the tumor site and predict the difference of the effects of different treatment schemes (Mahdi et al., 2025; Samathoti et al., 2025). Generally speaking, AI has upgraded nano-medicine from a static and empirical mode to a data-driven system, which can flexibly adjust between treatment intensity and bone repair needs by combining prediction, multi-objective decision-making and real-time feedback to help realize functional bone reconstruction.

## Discussion

Although AI-driven photoresponsive nano-platform provides a systematic way for the combination of osteosarcoma treatment and bone regeneration, there are still many problems in clinical application. This strategy needs multi-modal data (imaging, biomarkers, immune status, photothermal kinetics), but at present, the data standard and real-time monitoring have not been fully established, which limits the stability and applicability of the model in different scenarios. Therefore, standardized postoperative reconstruction datasets and high-precision digital twin models are particularly critical for multi-objective optimization and personalized prediction. Furthermore, issues such as data acquisition latency, sensor calibration, interoperability with existing clinical workflows, and regulatory validation pathways should be considered in future translational development. Addressing these practical aspects will be essential for clinical deployment.

The closed-loop control of illumination, drug release and immunomodulation timing also needs strong interpretability and security. Black-box models are difficult to pass supervision and ethical review, and may also affect the supervision of doctors. Therefore, we need the explainable AI framework to provide transparent reasons and practical support for each parameter adjustment, so as to improve traceability, trust and regulatory feasibility.

In addition to the combination of technology, AI-driven nanotherapy also represents a change in concept-from focusing on eliminating tumors to focusing on functional reconstruction. By embedding the loop of “perception-decision-execution-feedback “ in the treatment platform, the treatment scheme can be dynamically adjusted, which can not only control the tumor, but also protect the regenerative microenvironment. In this way, the evaluation of the therapeutic effect is not only to see whether the tumor has shrunk in the short term, but also to consider the long-term tissue repair and functional recovery. With the maturity of multi-modal sensing technology and interpretable multi-objective algorithm, the future evaluation criteria may consider both anti-cancer effect and regenerative sustainability, thus promoting accurate functional reconstruction in osteosarcoma treatment.

The left panel shows the therapeutic–regenerative paradox between tumor ablation and bone regeneration. The middle panel presents an AI-integrated nanoplatform that dynamically adjusts light irradiation and drug release by integrating multimodal data, including imaging, temperature, biomarkers, and immune status. The right panel illustrates a closed-loop adaptive framework (“Perception–Decision–Execution–Feedback”) that balances tumor eradication with functional bone reconstruction. This figure was created with BioRender (app.biorender.com).
